# Relationship Between Personality Disorders Scales, Pathological Personality Traits, and Six Domains of Functioning in Sample With Alcohol Use Disorder

**DOI:** 10.3389/fpsyt.2020.00498

**Published:** 2020-06-05

**Authors:** Jeļena Koļesņikova, Viktorija Perepjolkina, Velga Sudraba, Kristīne Mārtinsone, Ainārs Stepens

**Affiliations:** ^1^Department of Health Psychology and Paedagogy, Psychology Laboratory, Faculty of Public Health and Social Welfare, Riga Stradiņš University, Riga, Latvia; ^2^Department of Sociology and Psychology, Faculty of Communications, Riga Stradiņš University, Riga, Latvia; ^3^Department of Nursing and Midwifery, Faculty of Public Health and Social Welfare, Riga Stradiņš University, Riga, Latvia; ^4^Department of Health Psychology and Paedagogy, Faculty of Public Health and Social Welfare, Riga Stradiņš University, Riga, Latvia; ^5^Centre for Military Medicine Research, Riga Stradiņš University, Riga, Latvia

**Keywords:** alcohol use disorder, functioning, Latvian Clinical Personality Inventory, personality disorders, pathological personality traits

## Abstract

**Background:**

Studies reveal a functional impairment in patients with personality disorders (PDs), but there is not enough information to form conclusions about this relation in patients with alcohol use disorder (AUD). The aim of this study was to investigate to what extent a personality disorders scales including pathological personality traits (PPTs) predict six domains of functioning in patients with AUD.

**Methods:**

In total, 48 patients with AUD diagnosis, who were treated in the psychiatric clinics, aged 20 to 65 years [M = 37.5; SD = 12.08; 12 (25%) females and 36 (75%) males], filled out the demographic questionnaire, WHO Disability Assessment Schedule 2.0 (WHODAS 2.0, Latvian version) and Latvian Clinical Personality Inventory (LCPI v2.1.). All respondents signed the informed consent form.

**Results:**

Stepwise regression analysis showed that PD Avoidant scale positively predicts impairment in Cognition and Getting along domains of functioning in AUD patients, but, on the PPTs level, it was found that Social withdrawal along with Irresponsibility and Guilt/Shame positively predict impairment in Cognition domain of functioning, and Social withdrawal along with Depressivity and Irresponsibility positively predict impairment in Getting along domain of functioning. The results of the study showed that PPT Orderliness negatively predicts impairment in Live activities domain of functioning. The PD Dependent scale and PPT Separation insecurity positively predict impairment in Participation domain of functioning.

**Conclusions:**

Obtained results add deeper insight into understanding of the relationship between personality disorders scales including pathological personality traits and six domains of functioning in patients with AUD.

## Introduction

According to the World Health Organization (further—WHO), more than three million people worldwide die as a result of alcohol use. Alcohol misuse accounts for more than 5% of the global disease burden (1). The use of alcohol in Europe's regions is the highest in the world and remains a serious public health problem, causing Alcohol Use Disorder (further—AUD) and the consequences that hinder a person's ability in key areas of life (2, 3). The incidence of AUD in the population aged 15 and over in Latvia in 2016 is 10.4%, which is 3.7% times higher compared to European regions as a whole (1). As noted in a study by the Latvian Center for Disease Prevention and Control in 2017, the number of first-time patients with alcohol dependence in absolute numbers was 1,634 patients, of whom 1,194 were men and 440 were women (4).

Functional consequences of AUD create high probability of impairing functioning in the major areas of life, such as interpersonal relationships, and contributes to disinhibition and feelings of sadness and irritability (5), lack of self-cognition and social competences (6, 7), and difficulty in dealing with everyday problems (8). The results of recent studies indicate that those with AUD have an impairment in the domains of Participation in the society, household, and work-related activities as well as cognitive functioning, and also, some functional impairment is recorded in the domains of Mobility and Self-care (9, 10).

Research has consistently shown that AUD patients have high rates of psychiatric comorbidity (11). AUD is characterized by clinical, genetic and neurophysiological heterogeneity that is closely related to other mental disorders (12), for instance, anxiety, depressive, bipolar disorders, schizophrenia, and personality disorders (further—PDs) (13–17). PDs are four times more common among addictive and psychiatric patients compared to the general population (14).

### AUD Comorbidity With Personality Disorders and Functioning Impairment

PDs are made up of a group of psychiatric disorders that all share a common feature of an enduring, maladaptive behavior pattern markedly deviating from social expectations from individual's culture, which is pervasive and inflexible, stable over time, and leads to distress or impairment (5). As concluded in studies, PDs in alcohol addicted patients prevail up to 58–78%, besides, in literature there are often mentioned B-cluster disorders, especially borderline and antisocial PD (3, 18, 19). However, alcohol-dependent individuals have a whole spectrum of PDs, including avoidable, paranoid, dependent, and other PDs (13).

It is known that individuals with any PDs had greater impairment in instrumental role functioning in the previous 30 days, as assessed by days out of role, productive role functioning, and social role functioning (odds ratios 1.5, 1.6, and 2.0, respectively), adjusting for sociodemographic characteristics and Axis I disorders. Having a Cluster B PDs were strongly associated with impairment in social role functioning (OR 2.7) (20), for instance, individuals with borderline PD are likely to experience marked functional impairment, particularly in the social domain (21). Other research findings confirm that schizotypal and borderline PDs were significantly related to impairment at work, in social relationships and at leisure (22). There is also evidence from longitudinal studies that PDs are associated with future impairment. On the one hand, research reveals functional impairment in patients with PDs, but on the other hand, there is not enough information to form conclusions about such relation in patients with AUD.

### DSM-5 Dimensional Approach to PDs Diagnosis

Researchers pointed to the need to revise the diagnostic approach to PDs concerning high rates of comorbidity, the heterogeneity, observed among patients with the same diagnosis, and poor grounding of the cut-off points for differential diagnosis (23, 24). There are developed several dimensional models of personality pathology (25), one such model is described in DSM-5 (DSM-5 Trait model) (5). In the III section of the DSM-5, there is offered an alternative hybrid approach to PDs diagnostics, which combines both the categorical approach, based on the determination of the severity of the combined personality dysfunction, and the dimensional approach that allows obtaining the unique personality trait profile of a particular individual in PDs diagnostics (25). In this model, PDs are “characterized by impairments in personality functioning and pathological personality traits” [(5), p.761]. Despite the slightly differing views of experts about which pathological personality traits (PPTs) are most relevant and characterise particular PD [see (5, 26)], this approach could be very useful in clinical practice.

### Pathological Personality Traits in Patients With AUD

Studies have shown that the most frequent PPTs associated with alcohol abuse are impulsivity, neuroticism, negative affectivity (27, 28) and narcissism (29). Intense anger, impulsivity, and mood swings are characteristic both in case of alcohol addiction and for borderline personalities (18). Given this latest and reasoned hybrid approach to PD diagnostics, it would be important to find out what is the relationship not only between PDs with functioning impairment for AUD patients, but also with PPTs.

### Pathological Personality Traits and Functioning Impairment in Patients With AUD

In the study (30) which explored the relationship between the personality traits and six domains of the WHO Disability Assessment Schedule, WHODAS 2.0 (further—WHODAS) (5) in patients with dual diagnosis, there were found significant correlations between all functional domains and Anxiousness and Depressivity. The domain of Cognition shows correlations with Perceptual Dysregulation and Distractibility, the domain of Participation in society shows moderate correlations with Unusual Beliefs and Anxiousness. The domains of Getting along with people and Life activities show moderate correlations with Anhedonia, Eccentricity, and Irresponsibility.

It can be concluded that the research of PDs in connection with the difficulty of functioning in people with AUD may lead to superficial and limited conclusions, if the traits of abnormal personality are not analyzed in addition.

The aim of this study was to investigate the extent to what PDs including PPTs scales predict six domains of functioning in patients with AUD.

## Methods

### Participants

It was a cross-sectional (simultaneous) study with the target group defined as patients in psychiatric hospitals who were diagnosed with F10.2: Alcohol Dependence Syndrome based on ICD-10 (31). Criteria for inclusion in the particular study were: AUD diagnosis (F10.2.); Latvian is the native language; aged past 18; signed informed consent to participate. Criteria for exclusion were: assigned disability group; diagnosed other mental disorders of Axis I (31); time after detoxification therapy less than 14 days.

From 180 patients of the psychiatric hospitals who signed informed consent to participate in the initial stage of validation study, 48 patients were recognized as meeting the above mentioned criteria and were selected for inclusion in the sample of this study. These patients were aged 20 to 65 years (M = 37.5; SD = 12.08), 12 (25%) were females and 36 (75%) were males. All patients included in the study were treated in the programs which are based on the Minnesota Model approach. None of those involved in the study had a diagnosis of PDs and behavioral disorders in adults (F60-F69) by ICD-10 (31).

### Measures

The survey packet consisted of 707 items in total divided in the three parts: 1) demographic questions (7 items), 2) the World Health Organization Disability Assessment Schedule (WHODAS 2.0) (36 items), and 3) the Latvian Clinical Personality Inventory version 2.1. (LCPI v2.1.) (664 items).

The first part included demographic questions about age, gender, native language, mental health, time after detoxification therapy, and disability group.

The second part was the WHODAS 2.0 (32), adapted in Latvia by Bērziņa, 2016 (33), which is a generic assessment instrument for health and disability, a tool to produce standardized disability levels and profiles, applicable across cultures, in all adult populations, directly linked at the level of the concepts to the ICF. It contains 36 items and six domains: Cognition, Mobility, Self-care, Getting along, Life activities, and Participation (Cronbach's alpha varied from 0.81 to 0.93). It measures the ability of functioning and daily activity over the last 30 days and difficulties or incapacity associated with dysfunction.

The third part was the preliminary version of the Latvian Clinical Personality Inventory version 2.1. (LCPI v2.1.) (34). LCPI v2.1. consists of 664 true-false items. In the present study, there were used only 10 scales of personality disorders (PDs) (Schizotypal, Schizoid, Paranoid, Histrionic, Narcissistic, Antisocial, Borderline, Avoidant, Dependent, and Compulsive) and 34 facet-level scales of PPTs. All the 10 scales of PDs and 34 PPT scales show acceptable to high internal consistency (for PDs scales Cronbach's alpha varied from 0.68 to 0.90 and for PPTs scales Cronbach's alpha varied from 0.67 to 0.90) (35).

### Data Collection

Data were collected from December 2016 to February 2017. All procedures were approved by the Riga Stradiņš University Ethical board. Data were collected by meeting each patient with AUD individually. All patients signed the informed consent form. During the testing session, patients filled out the demographic questionnaire, LCPI version 2.1, WHODAS 2.0. The confidentiality of respondents was fully respected in data collection and processing. Only valid protocols (based on the LCPI Validity scales) were used in this study. This study formed part of a LCPI-v3 development and validation research carried within the framework of Latvian National Research Programme Biomedicine for Public Health (BIOMEDICINE) 2014–2017 (sub-project Nr.5.8.2).

### Data Analysis

For the processing and analysis of the data, SPSS software v.20.0 was used. To investigate the relationships between WHODAS domains, PDs scales and PPTs, the Spearman's rank correlation method was used. Simple and multiple stepwise linear regression analysis was conducted to find out which PDs and PPTs are predictive for functioning impairment. *R*^2^ change effect sizes were interpreted by Cohen (36) conventions (change effects of .01, .06, and .14 were interpreted as small, medium, and large, respectively). For power analysis (to determine the probability of detecting a “true” effect when it exists), G*Power version 3.1.9.2. (37) was used.

## Results

In the first stage of data analysis, there were analyzed relationships between WHODAS domains both with PDs and PPTs scales. The obtained results are shown in the **Tables 1** and **2**.

**Table 1:** Spearman's rank correlation coefficients between personality disorders scales and six domains of functioning.

WHODAS 2.0.	Paranoid	Schizoid	Schizotypal	Antisocial	Borderline	Histrionic	Narcissistic	Avoidant	Dependent	Obsessive-Compulsive
Cognition	.10	.57**	.39*	.15	.49**	−.14	−.02	.65**	.50**	−.08
Mobility	.15	.14	.27	.21	.23	.04	.07	.16	.26	−.20
Self-care	.09	−.06	.16	.19	.20	.23	.14	.05	.20	−.23
Getting along	.11	.58**	.32*	.17	.41**	−.23	−.08	.62**	.38*	−.12
Life activities	−.08	−.01	.15	.19	.10	.13	−.06	.11	.22	−.39*
Participation	.22	.27	.40**	.27	.47**	.11	.12	.39**	.48**	−.13

*p < 0.05, **p < 0.01.

**Table 2:** Spearman's rank correlation coefficients between pathological personality traits and six domains of functioning.

Pathological personality traits	Domains of functioning						Pathological personality traits	Domains of functioning					
Subordination	Cognition	Mobility	Self-care	Getting along	Life activities	Participation	Antagonism	Cognition	Mobility	Self-care	Getting along	Life activities	Participation
Submissiveness	.24	.21	.21	.23	.11	.24	Callousness	.16	.20	.17	.14	.14	.26
Separation insecurity	.22	.36*	.21	.13	.30	.29*	Hostility	.18	.19	.07	.21	.07	.24
Low self-esteem	.16	.29*	.20	.13	−.04	.12	Manipulativeness	.06	.17	.25	−.00	.17	.18
Guilt/shame	.30*	.11	.16	.25	.28	.33*	Will to power	−.00	.13	.19	−.00	.13	.13
							Grandiosity	−.10	.11	.14	−.10	−.09	.07
							Histrionism	.15	−.00	.00	.05	.05	.09
							Attention seeking	.02	.06	.18	−.11	.08	.16
							Deceitfulness	.11	.02	.14	.30*	−.02	.06
**Negative Emotionality**							**Disinhibition**						
Emotional lability	.39**	.19	.12	.24	.15	.47**	Impulsivity	.15	−.04	−.01	.12	.12	.24
Anxiousness	.43**	.29*	.25	.31*	.32*	.46**	Recklessness	−.04	.05	.01	−.01	−.03	.05
Depressivity	.45**	.29*	.28	.44**	.06	.42**	Irresponsibility	.54**	.29*	.28	.46**	.28	.39**
Distrustfulness	.29*	.07	.04	.22	−.15	.26	Aggression	.20	.17	.18	.15	.08	.30*
**Detachment**							**Psychoticism**						
Social withdrawal	.56**	.10	−.15	.55**	−.01	.20	Eccentricity	.24	.21	.21	.23	.11	.24
Social detachment	.49**	.13	−.05	.55**	.02	.23	Unusual perceptions	.22	.36*	.21	.13	.30	.29*
Anhedonia	.40**	.14	−.08	.36*	−.02	.20	Unusual beliefs	.16	.29*	.20	.13	−.04	.12
Intimacy avoidance	.18	.10	.14	.16	−.12	.17	Cognitive dysregulation	.20	.11	.18	.09	−.10	.17
Restricted affectivity	−.00	.22	−.06	−.01	.01	−.12	Dissociation proneness	.59**	.37**	.17	.46**	.31	.44**
							Suspiciousness	.04	.15	.13	.02	−.02	.22
							Self-Harm	.08	.22	.10	−.01	−.01	.25
**Compulsivity**													
Perfectionism	.04	−.06	−.18	.02	−.27	−.07							
Orderliness	−.16	−.13	−.19	−.22	−.40**	−.15							

*p < 0.05. **p < 0.01.

It was found that 4 of 10 PDs (Paranoid, Antisocial, Histrionic, and Narcissistic) show no statistically significant correlations with any of six domains of functioning. Obsessive-Compulsive PD is statistically significantly negatively associated only with WHODAS domain Life activities; Schizotypal, Borderline, Avoidant, and Dependent PDs are all statistically significantly positively associated with three domains of WHODAS: Cognition, Getting along and Participation, but Schizoid PD is positively associated with two of six WHODAS domains: Cognition and Getting along (see **Table 1**).

In **Table 2**, correlations between 34 PPTs and 6 WHODAS domains are presented. As it can be seen, 17 of 34 PTTs (Submissiveness, Intimacy avoidance, Restricted affectivity, Perfectionism, Callousness, Hostility, Manipulativeness, Will to power, Grandiosity, Histrionism, Attention seeking, Impulsivity, Recklessness, Eccentricity, Cognitive dysregulation, Suspiciousness, and Self-Harm) show no statistically significant correlation with any of WHODAS domain in the group of patients with AUD. WHODAS domain Cognition is positively associated with all four traits from Negative Emotionality domain (Emotional lability, Anxiousness, Depressivity, Distrustfulness), three of five traits from the Detachment domain (Social withdrawal, Social detachment, and Anhedonia), with Irresponsibility from Disinhibition domain and with Dissociation proneness from Psychoticism domain. WHODAS domain Mobility is positively associated with eight PPTs: Separation insecurity, Low self-esteem, Anxiousness, Depressivity, Irresponsibility, Unusual perceptions, Unusual beliefs, Dissociation proneness. Six PPTs (Anxiousness, Depressivity, Social withdrawal, Social detachment, and Anhedonia, Deceitfulness, Irresponsibility, Dissociation proneness) is significantly positively related to Getting along domain; two, Anxiousness (positively), Orderliness (negatively), to Life activities domain, and nine PPTs, Separation insecurity, Guilt/shame, Emotional lability, Anxiousness, Depressivity, Irresponsibility, Aggression, Unusual perceptions, and Dissociation proneness, to Participation domain. No statistically significant correlation was found with Self-care domain of WHODAS (see **Table 2**).

Taking into account the significant number of PDs scales and PPTs scales found to be associated with WHODAS domains, at the next stage of data analysis, series of stepwise regression analysis was conducted to find out which PDs and PPTs could be predictive for functioning impairment. In each regression, the WHODAS domains score was included as a dependent variable, and PDs and PPTs scales found to be statistically significantly associated with particular WHODAS domain (see **Table 3**), were entered as independent variables. Seven separate regression analyses were performed, first three analyses with PDs scales as independent variables, and then four analyses with PPTs scales as independent variables.

**Table 3:** Stepwise regression analysis for six domains of functioning as the dependent variables and personality disorders scales (PDs) and pathological personality traits (PPTs) scales as independent variables in group of patients with AUD.

	β	F	R^2^	ΔR^2^ (Adjusted R^2^)		β	F	R^2^	ΔR^2^ (Adjusted R^2^)
**Cognition** (DV*)					**Cognition** (DV*)				
Step 1		37.89**	.45	.45 (.44)	Step 1		27.91**	.38	.38 (.37)
PD Avoidant	.67**				PPT Social withdrawal	.62**			
					Step 2		24.92**	.53	.14 (.51)
					PPT Social withdrawal	.49**			
					PPT Irresponsibility	.40**			
					Step 2		22.82**	.61	.08 (.58)
					PPT Social withdrawal	.52**			
					PPT Irresponsibility	.34**			
					PPT Guilt/shame	.29*			
**Getting along** (DV*)					**Getting along** (DV*)				
Step 1		42.23**	.47	.47 (.46)	Step 1		27.17**	.37	.37 (.36)
PD Avoidant	.69**				PPT Social withdrawal	.61**			
					Step 2		20.50**	.48	.10 (.46)
					PPT Social withdrawal	.50**			
					PPT Depressivity	.34*			
					Step 3		16.08**	.53	.04 (.49)
					PPT Social withdrawal	.46*			
					PPT Depressivity	.25*			
					PPT Irresponsibility	.24*			
					**Life activities** (DV*)				
					Step 1		5.95*	.14	.14 (.11)
					PPT Orderliness	−.37*			
**Participation** (DV*)					**Participation** (DV*)				
Step 1		12.93**	.22	.22 (.20)	Step 1		15.33**	.25	.25 (.24)
PD Dependent	.47**				PPT Separation insecurity	.50**			

*p < 0.05. **p < 0.01. DV*, dependent variable.

### Cognition Domain

The results of the stepwise regression analyses indicated that PD Avoidant scale positively predicted impairment in the Cognition domain and explained 45% of the total variance. When PPTs were analyzed as independent variables, in step 1 of the model, the PPT Social Withdrawal explained 38% of the total variance in Cognition domain. In step 2, the PPT Social Withdrawal along with PPT Irresponsibility explained 53% of the total variance in Cognition domain, and in step 3, the PPT Social Withdrawal along with PPT Irresponsibility and PPT Guilt/Shame explained 61% of the total variance in Cognition domain (see **Table 3**).

### Getting Along Domain

The results indicated that PD Avoidant scale positively predicted impairment in Getting Along domain and explained 47% of the total variance in this domain. When PPTs were analyzed as independent variables, in step 1 of the model, the PPT Social Withdrawal explained 37% of the total variance in Getting Along domain. In step 2, the PPT Social Withdrawal along with PPT Depressivity explained 48% of the total variance in Getting Along domain, and, in step 3, the PPT Social Withdrawal along with PPT Depressivity and PPT Irresponsibility explained 53% of the total variance in Getting Along domain (see **Table 3**).

### Participation Domain

The results indicated that PD Dependent scale positively predicted impairment in Participation domain and explained 22% of the total variance in the domain. When PPTs were analyzed as independent variables, in step 1 of the model, the PPT Separation Insecurity positively predicted impairment in Participation domain and explained 25% of the total variance in the domain (see **Table 3**).

### Life Activities Domain

Finally, it was found that PPT Orderliness negatively predicted impairment in the Life Activities domain and explained 14% of the total variance of this domain (see **Table 3**).

Based on performed Post Hoc power analysis for evaluation for 1) the deviation of R^2^ from zero for total model and 2) the increase of R^2^ in the multiple regression model, it was found that almost in all cases presented in **Table 3**, the power of the performed test was higher than 0.80, mostly power indices were from 0.95 to 0.99. Slightly lower than acceptable (0.80), it was for R^2^ change for Orderliness in the last step of the model, when predicting Life Activities domain (power of that test was 0.78), and not acceptable (0.50) for R^2^ change for Irresponsibility, when predicting Getting along domain.

## Discussion

To achieve the aim of this study, it was necessary to identify the extent to which LCPI v2.1. scales of PDs and PPTs predict impairments in functional domains in patient group with AUD.

Results of the study show that Avoidant PD scale positively predicts impairments in the Cognition domain (R^2^ = .45) and Getting along domain (R^2^ = .47) in patients with AUD. This means that from AUD patients who have a high manifested pattern of Avoidant personality, one can expect that they will have impairments in such cognitive functions as concentrating, remembering, problem-solving, learning and communicating, and difficulties with interactions with other people.

Analyzing what PPTs, relevant to Avoidant PD, are predictive to these two functional domains, it was found that: 1) three PPTs: Social Withdrawal (ΔR^2^ =.38) [which, according to DSM-5, is defined as “preference for being alone to being with others, reticence in social situations, avoidance of social contacts and activity and lack of initiation of social contact” [(5), p. 831)], Irresponsibility (ΔR^2^ =.14) [which is characterized as “disregard for and failure to honour financial and other obligations or commitments, lack of respect for and lack of follow-through on agreements and promises” [(5), p. 765)], and Guilt/Shame (ΔR^2^ =.08) positively predicted functional difficulties in the Cognition domain; the effect size when all three variables were included in the model, was R^2^ = .61, which is characterized as large effect (based on Cohen (38), R^2^ = 0.015 denotes a small effect, R^2^ = 0.125 a medium effect and R^2^ = 0.255 a large effect); 2) Social Withdrawal (ΔR^2^ = .37), Depressivity (ΔR^2^ = .10), and Irresponsibility (ΔR^2^ = .04) most accurately and consistently predict functional difficulties in Getting along domain (but it must be mentioned that in this model, the personality trait Irresponsibility does not have sufficient strength for further interpretation). These findings imply that for AUD patients who have a highly manifested pattern of social withdrawal, feelings of inadequacy, and hypersensitivity to negative evaluation, it is possible to predict functional difficulties in Cognition domain, but for patients with highly manifested pattern of social withdrawal in combination with Depressivity, which is characterized as feelings of being down, hopeless, and pessimism about the future [(5), p. 779], it is possible to predict difficulties in understanding, communicating and interacting with other people.

Knowing which PPTs are predictive for impairment in Cognition domain and Getting along domain, specialists can provide more individual approach in treatment of patients with AUD. For example, they can anticipate that patients with high scores of social withdrawal may be “bad” candidates for various types of treatment that will require group work. Specialists treating such patients, especially in psychoeducational programs, must take into account that for such an individual it could be difficult not only to take part in the group work, but also to understand, analyze, and remember discussed material. So, when working with such patients, it could be useful to use methods that reduce intolerable feelings, especially in the initial stages of treatment, when the highest rates of dropouts mostly occur.

It has been also found that PD that most likely predicts difficulties in Participation domain is Dependent PD scale (R^2^ = .22) that corresponded to medium effect size. The possibility to analyse the results in greater detail allows to find out that the most significant predictor of difficulties in Participation domain is PPT Separation insecurity (R^2^ = .25), which is related to “fears of rejection by and/or separation from significant others, associated with fears of excessive dependency and complete loss of autonomy” [(5), p. 767] and is one of the most relevant traits of Dependent PD. It means that AUD patients who have a high manifested pattern of submissive and clinging behavior related to an excessive need to be taken care of, tend to have functional difficulties in Participation domain that include community activities. So it could be suggested that, when working with individuals who have pronounced personality trait Separation insecurity, the specialist must take into account that the individual will have difficulties with participation and co-responsibility, and the dependent individual will expect instructions, advice, and will expect the specialist to act authoritatively, on the directive way. Such patients often indulge in a specialist and follow his/her instructions not because the individual believes in those instructions and not for the sake of improvement, but so that the specialist would not leave them. They are often afraid of improvement, because then they will have to stop treatment, and thus the relationship with the specialist.

Finally, it was found that PDs do not predict domain of Live activities, but, looking at the obtained results on the traits level, it was found that PPT Orderliness negatively predict functional difficulties in this domain (R^2^ = .14). This means that for AUD patients who have a high manifested Orderliness, which is related to preoccupation with details, organization, and order, it is possible to predict that functional difficulties will be somewhat smaller in Live activities domain that includes difficulty with day-to-day activities (i.e., the ones people do on most days, including those associated with domestic responsibilities, leisure, work, and school).

Generally, the results are consistent with results of previous studies and more precisely explain the problems of functioning of the patients of the AUD group in terms of both PDs and PPTs. For example, it was shown (30) that people with dual diagnoses have difficulty developing a meaningful social life and have anhedonia and anxiety.

Both alcohol abuse and PDs are difficult to treat and are a challenge for healthcare providers. PDs cause difficulties in developing therapeutic relationships, difficulty in attending, and more frequent relapses (drop-outs). Moreover, based on results of this study, it can be concluded that assessment of PPTs could constitute an important target of relapse prevention and treatment programmes in patients' group with AUDs, as evidenced by medium to large effect sizes (R^2^ ranging from 0.14 to 0.61).

AUD persons may have problems with the self-care and mobility functioning, but, in this study, it was not revealed that there would be a relation between self-care and mobility and PDs or PPTs, which was discovered in other studies for patients with AUD. These results are partially consistent with those reported by Keeley et al. (39) with a clinical sample. Researchers found the facets to be most closely related to the domains of understanding and communicating, getting along with people, and participation in society. Similarly, in the present sample of AUD patients, we found that the personality traits are related mainly to aspects of the interpersonal relationships of these patients, which constitutes validity evidence consistent with that shown by other authors.

To summarize, it is important to pay attention that in the practice of individual analysis of patient traits, PPTs can form a unique profile and combine a number of variations, which indicates that, when analyzing the types of PDs based on a categorical approach, it is risky to ignore the unique PPTs of patient profile, as this may cause errors in treatment planning and forecast.

### Limitations

It is known that cross-sectional studies have limitations to predictions. For example, the results could differ in another time period; therefore, it would be necessary to conduct a longitudinal study. It must be acknowledged that there is disadvantage in the fact that the study included only patients who had turned to a specialist for help and did not include those who had turned to a specialist or visited private medical institutions. Another important factor is the duration of the individual's illness with the AUD, which was not controlled in this study, but as it is known, has a negative impact on the various areas of functioning (40). Studies often show a high percentage of PDs in the group of patients with substance use disorder, and B-cluster PDs are more common (3, 18, 19, 41), but there were no patients with PD diagnosis in this sample, which most likely reflects the limitation of the patient selection procedure on the one hand, since inclusion in the sample served as an AUD diagnosis criterion and patients with comorbid Axis I disorders were not included, which could potentially conceal a large population of patients with PDs as well. Moreover, it is necessary to note some difficulties in the diagnosis of comorbid disorders. It is possible that inclusion of all patients with comorbid mental disorders and mandatory additional diagnostics of the mental status of patients, as well as more sufficient statistical analysis with control of confounded variables can provide more clear and reliable results.

There were some more limitations regarding the sample size and composition. First, it represents only Latvian speaking part of AUD patients, second, sample size is small, which limits the possibility to generalize findings of the study. Increasing of the sample size might have improved the estimated reliability of the scores by increasing their variability (42) and the power of the statistical methods employed. Despite this limitation, authors of this study believe that their sample size was sufficient for successful implementation of the conducted statistical analyses, which was approved by the results of the Power analysis.

Due to the small sample size, the gender factor was not taken into account in this study, although it was shown that there are gender differences in personality traits, for example, obsessive-compulsive, hysterical, and antisocial PDs prevail among women (43).

Also, the limitations concern the possibility that functioning might not be adequately assessed in this type of patients when using self-report as the test measure. Optimal use of multisource data, such as family members' reports or register data, could improve the prediction of behavior in the clinical assessment of psychopathology (30, 44).

## Conclusions

This study revealed that the obtained results add deeper insight into understanding of the relationship between PDs scales, PPTs, and functioning domains in patients with AUD. The avoidant and dependent PDs scales predict impairment in functioning domains in AUD patients but, on the PPT level, there was found additional useful information. Social withdrawal, Irresponsibility, Guilt/Shame, Separation insecurity, and Depressivity predict impairment in everyday functioning. These results suggest that not only PDs scales but also evaluation of PPTs should be taken into consideration by practitioners for planning more effective approaches in treatment of alcohol addicted patients.

## Data Availability Statement

The raw data supporting the conclusions of this article will be made available by the authors, without undue reservation, to any qualified researcher.

## Ethics Statement

All patients provided their written informed consent to participate in this study. The study was reviewed and approved by the Rīga Stradiņš University Research Ethics Committee and by the Riga Psychiatry and Narcology Centre.

## Author Contributions

JK, VP, VS, KM, and AS have contributed to the conception and design of the study “Relationship between personality disorders scales, pathological personality traits and six domains of functioning in sample with alcohol use disorder”. JK and VS organized the database. JK and VP performed the statistical analysis. JK wrote the first draft of the manuscript. JK and VS wrote sections of the manuscript. All authors contributed to the manuscript revision and have approved the submitted version.

## Funding

The work was supported by Grants No. 5.8.2 of the National Research Programme of Latvia (Biomedicine, 2014–2017) and Nr. 48- 23/2017/0452. 2017–2020, project “The Development of Digitalized Personality Assessment System”.

## Conflict of Interest

The authors declare that the research was conducted in the absence of any commercial or financial relationships that could be construed as a potential conflict of interest.
